# Ascorbate peroxidase plays an important role in photoacclimation in the extremophilic red alga *Cyanidiococcus yangmingshanensis*

**DOI:** 10.3389/fpls.2023.1176985

**Published:** 2023-06-02

**Authors:** Han-Yi Fu, Ming-Wei Wang

**Affiliations:** Department of Biological Sciences, National Sun Yat-sen University, Kaohsiung, Taiwan

**Keywords:** reactive oxygen species, superoxide dismutase, monodehydroascorbate reductase, dehydroascorbate reductase, glutathione reductase, F_v_/F_m_

## Abstract

**Introduction:**

Acidothermophilic cyanidiophytes in natural habitats can survive under a wide variety of light regimes, and the exploration and elucidation of their long-term photoacclimation mechanisms promises great potential for further biotechnological applications. Ascorbic acid was previously identified as an important protectant against high light stress in *Galdieria partita* under mixotrophic conditions, yet whether ascorbic acid and its related enzymatic reactive oxygen species (ROS) scavenging system was crucial in photoacclimation for photoautotrophic cyanidiophytes was unclear.

**Methods:**

The significance of ascorbic acid and related ROS scavenging and antioxidant regenerating enzymes in photoacclimation in the extremophilic red alga *Cyanidiococcus yangmingshanensis* was investigated by measuring the cellular content of ascorbic acid and the activities of ascorbate-related enzymes.

**Results and discussion:**

Accumulation of ascorbic acid and activation of the ascorbate-related enzymatic ROS scavenging system characterized the photoacclimation response after cells were transferred from a low light condition at 20 μmol photons m^–2^ s^–1^ to various light conditions in the range from 0 to 1000 μmol photons m^–2^ s^–1^. The activity of ascorbate peroxidase (APX) was most remarkably enhanced with increasing light intensities and illumination periods among the enzymatic activities being measured. Light-dependent regulation of the APX activity was associated with transcriptional regulation of the chloroplast-targeted APX gene. The important role of the APX activity in photoacclimation was evidenced by the effect of the APX inhibitors on the photosystem II activity and the chlorophyll a content under the high light condition at 1000 μmol photons m^–2^ s^–1^. Our findings provide a mechanistic explanation for the acclimation of *C. yangmingshanensis* to a wide range of light regimes in natural habitats.

## 1 Introduction

Cyanidiophyceae are a class of unicellular red algae that diverged very early from the ancestral lineage of red algae (Yoon et al., 2004). A recent taxonomic classification reveals that Cyanidiophyceae comprise three orders of acidothermophilic red algae (Galdieriales, Cyanidiales, and Cyanidioschyzonales) and one order of mesophilic red algae (Cavernulicolales) (Park et al., 2023). The acidothermophilic cyanidiophytes can grow under relatively low pH (pH 0–5) and high temperature (up to 65°C) conditions and are tolerant to high salinities (Albertano et al., 2000; Van Etten et al., 2022). They can grow massively with low risk of cross-contamination in the open culture combined with acidic seawater condition due to their ability to live under multiple extreme conditions (Hirooka et al., 2020). Thanks to the relatively simple and nonsterile culture condition, acidothermophilic cyanidiophytes thus achieves a competitive advantage over other microalgae for biotechnological and industrial applications. Various potential applications have been proposed for these polyextremophilic red algae. Several cyanidiophytes demonstrate a high capacity to remove toxic hexavalent chromium in acidic and neutral conditions (Cho et al., 2023). *Galdieria* spp. can utilize more than 50 organic compounds as carbon sources, and their use for removal of organic carbons in wastewater treatment and for recovery of rare metal elements has been reported (Gross and Schnarrenberger, 1995; Minoda et al., 2015; Henkanatte-Gedera et al., 2017). The genetic engineering approaches for *Galdieria* spp. and *Cyanidioschyzon merolae* are available for investigating potential usage in biofuel production and other biotechnological applications (Pancha et al., 2021; Hirooka et al., 2022).

Biogeographic studies on the distribution of acidothermophilic cyanidiophytes indicate that these polyextremophiles are adapted to a great variety of light regimes (Brock and Brock, 1971; Ciniglia et al., 2004; Toplin et al., 2008; Hsieh et al., 2015). The light conditions of their microhabitats range from an extreme low light intensity (e.g. in endolithic and interlithic habitats) to a sunlight level (e.g. in stream and open ponds). A distance-based redundancy analysis revealed that the light regime did not affect the community structure of Cyanidiophyceae species in the acidic habitats (Hsieh et al., 2018). Although these cyanidiophytes were found under a great variety of light conditions, they appeared to be sensitive to high illumination in some cases. A decreased growth rate and a yellowish color were observed when the *C. merolae* cells were transferred to a relatively high light condition at 200 μmol photons m^–2^ s^–1^, suggesting that these cells suffered from high light stress (Minoda et al., 2004). A similar observation was also reported in *Galdieria partita*, cells of which became bleached and did not grow when they were transferred from a low light condition at 20 μmol photons m^–2^ s^–1^ to a high light condition at 300 μmol photons m^–2^ s^–1^ (Fu et al., 2020). Interestingly, inhibition of photosynthesis under high illumination was observed in natural populations of *Cyanidium caldarium* acclimated to low light conditions for 1 month, whereas none of the populations acclimated to high light conditions showed such inhibition (Doemel and Brock, 1971). The contrasting responses of high light- and low light-acclimated *C. caldarium* suggest that photoprotective processes are crucial in long-term acclimation of cyanidiophytes to high light conditions.

Accumulation of ascorbic acid was recently identified as an important photoprotective process of *G. partita* against high light stress under mixotrophic culture with glucose supplemented (Fu et al., 2020). Scavenging of reactive oxygen species (ROS) produced by photosystems is one important function exerted by ascorbic acid during the photoprotective process. Ascorbic acid can directly quench singlet oxygen (^1^O_2_), superoxide ( ${\text{O}}_{2}^{\;•-}$ ), and hydroxyl radical (OH^•^), as well as participate in an enzymatic ROS scavenging system (Asada, 2006; Pinnola and Bassi, 2018). In this enzymatic system, superoxide dismutase (SOD) catalyzes conversion of ${\text{O}}_{2}^{\;•-}$ to hydrogen peroxide (H_2_O_2_), which then reacts with ascorbic acid through ascorbate peroxidase (APX) to produce monodehydroascorbate (MDHA) radical and water. Ascorbic acid can be regenerated from reduction of MDA through MDA reductase (MDHAR) or from spontaneous disproportionation of MDA into dehydroascorbate (DHA) and ascorbic acid. DHA can be further reduced to ascorbic acid *via* the reaction with reduced glutathione and catalyzed by DHA reductase (DHAR). If this enzymatic ROS scavenging system plays an important role in photoacclimation, it is anticipated that the cellular content of ascorbic acid and the activities of ascorbate-related enzymes increase to counteract photodamage. Indeed, the enhanced activities of SOD, APX, and glutathione reductase (GR) together with the increased amount of ascorbic acid were reported in plants in response to high illumination (Grace and Logan, 1996). Whether activities of these ROS scavenging enzymes and antioxidant regenerating enzymes are enhanced in cyanidiophytes under high illumination awaits more extensive exploration.

In this work, *Cyanidiococcus yangmingshanensis* was chosen for the investigation of the role played by the ascorbate-related enzymatic ROS scavenging system in photoacclimation. *G. partita* and *C. yangmingshanensis* (previously misidentified as *G. maxima*) were two dominant Cyanidiophyceae species living in the Tatun Volcano Group area in Taiwan (Hsieh et al., 2015; Liu et al., 2020). *C. yangmingshanensis* is phylogenetically distant from *Galdieria* spp. and close to *C. merolae* based on the comparative analysis through genomic, physiological, and morphological traits (Liu et al., 2020). *C. yangmingshanensis*, like *C. merolae*, is an ecologically obligate photoautotroph and cannot use external organic carbons at concentrations up to 200 mM for heterotrophic growth (Moriyama et al., 2015; Liu et al., 2020). Relatively high abundance of *C. yangmingshanensis*, compared to that of *G. partita*, was found under diverse light regimes from low to high light conditions in natural habitats (Hsieh et al., 2015; Hsieh et al., 2018). Cells of the isolated *C. yangmingshanensis* strain could photoautotrophically proliferate when they were transferred from low illumination at 20 μmol photons m^–2^ s^–1^ to high illumination at 300 μmol photons m^–2^ s^–1^, whereas cells of the isolated *G. partita* strain could proliferate only with glucose supplemented upon the same light treatment (Fu et al., 2020; Liu et al., 2020). The ability of *C. yangmingshanensis* to be acclimated to a broad range of light irradiance without the intervention of metabolites derived from supplemented organic carbons makes *C. yangmingshanensis* a more advantageous microalga than *G. partita* for the identification of the long-term photoacclimation mechanism.

The aim of this work was to investigate the involvement of the ascorbate-related enzymatic ROS scavenging system in photoacclimation as well as to identify the key enzyme regulated by light and crucial in photoacclimation of *C. yangmingshanensis*. The photoacclimation response was characterized by cell proliferation, changes in the cellular content of photosynthetic pigments, and changes in the F_v_/F_m_ level. Furthermore, light-dependent regulation of the enzymatic ROS scavenging system was assessed by the changes in the cellular content of ascorbic acid and in the activities of SOD, APX, MDHAR, DHAR, and GR. The importance of the enzymatic ROS scavenging system in photoacclimation was evaluated using inhibitors of an enzyme that was shown to be the most tightly regulated in a light-dependent manner.

## 2 Materials and methods

### 2.1 Culture conditions

The *C. yangmingshanesis* strain THAL066 was obtained from Tung-Hai Algal Lab Culture Collection at Tunghai University, Taichung, Taiwan (https://sites.google.com/view/algae-molecular-ecology-lab/tung-hai-algal-lab-culture-collection). Cells were grown in modified Allen’s (MA) medium (pH adjusted to 2.0 with H_2_SO_4_) (Minoda et al., 2004) at 40°C with shaking at 150 rpm under continuous cool-white fluorescent light (20 μmol photons m^−2^ s^−1^). Liquid cultures were diluted every month to ensure that cells were grown in the exponential phase. For the photoacclimation experiment, cells were harvested and resuspended in MA medium to the cell density equivalent to 2×10^7^ cells mL^−1^. Cells were grown at 40°C with shaking at 150 rpm under continuous white LED light conditions. Light intensity was set to 0 μmol photons m^−2^ s^−1^ for the dark condition, 20 μmol photons m^−2^ s^−1^ for LL, 200 μmol photons m^−2^ s^−1^ for ML, and 1000 μmol photons m^−2^ s^−1^ for HL. Erlenmeyer flasks were wrapped with aluminum foil to ensure that cells in the dark condition were completely devoid of light.

### 2.2 Quantification of chlorophyll (Chl) *a*, carotenoids, and phycocyanin

Quantification of Chl *a* was described previously (Fu et al., 2020) with minor modifications. In brief, 1.5 mL of cells were harvested by centrifugation at 16,000 ×g for 2 min at 4°C. The harvested cells were resuspended in DMSO and incubated at 65°C for 10 min. After centrifugation, the supernatant was measured using a JASCO V-630 UV-visible spectrophotometer (JASCO). The Chl *a* concentration was estimated using the following equations: Chl *a* (μg/mL) = (A_665.1 nm_ – A_750 nm_) ÷ 0.0834 (Fu et al., 2020). The Chl *a* content was calculated as the Chl *a* concentration multiplied by the cell density as estimated using a hemocytometer.

Extraction of pigments for quantification of carotenoids was performed according to the method of Cunningham et al. (2007) with modifications. The harvested cells were resuspended in 300 μL of acetone: methanol 7:2 (v/v) with 0.07 g sea sand and then vortexed at the speed of 2,500 rpm for 15 min at 4°C. After centrifugation, 200 μL of the extract was transferred to another tube, and the pellet was resuspended in 300 μL of ethyl acetate and then vortexed in the same condition as mentioned. The second extract (300 μL) was combined with the first and added with 400 μL of deionized water. Phase separation was conducted by gentle mixing followed by centrifugation at 16,000 g for 2 min at 4°C, and the upper phase was transferred to a new tube for HPLC analysis on a Prominence LC-20AT HPLC system equipped with an SPD-20A UV/Vis detector (Shimadzu). The HPLC analysis procedure for quantification of zeaxanthin, β-cryptoxanthin and β-carotene was described previously (Fu et al., 2020). The cellular content of individual carotenoids was calculated as the molar ratio of carotenoids to Chl *a* multiplied by the Chl *a* content.

Phycocyanin was extracted and estimated according to the methods described by Eisele et al. (2000) with modifications. In brief, cells were centrifuged at 8,000 ×g for 5 min at 4°C. The harvested cells were washed twice with 100 mM sodium phosphate buffer (pH = 6.8) and then resuspended with 100 μL of the same buffer to the cell density equivalent to 4×10^9^ cells/mL. The cells were disrupted with 0.08 g of glass beads (0.5 mm in diameter) using a Bullet Blender Storm bead-mill homogenizer (Model BBY24M, Next Advance) at the speed set to 10 for 5 min at 4°C. After centrifugation at 20,000 ×g for 15 min at 4°C, 50 μL of supernatant was diluted with 150 μL of 100 mM sodium phosphate buffer and measured. The concentration of phycocyanin was calculated using the following equations: phycocyanin (mg/mL) = (A_620 nm_ – 0.474 ×A_652 nm_) ÷ 5.34 (Moon et al., 2014). To eliminate the scattering effect in the sample, the A_620 nm_ and A_652 nm_ values were corrected based on two isosbestic points of phycocyanin at 470 nm and 730 nm using the following equations: corrected A_620 nm_ = A_620 nm_ – (A_470 nm_ − A_730 nm_) ÷ 260 × 110 – A_730 nm_, corrected A_652 nm_ = A_652 nm_ – (A_470 nm_ − A_730 nm_) ÷ 260 × 78 – A_730 nm_.

### 2.3 Quantification of ascorbic acid

The ascorbic acid content was estimated using a colorimetric method described by Shigeoka et al. (1987) with modifications. Harvested cells were washed twice in the MA medium at 4°C and then resuspended in 100 μL of 5% (w/v) metaphosphoric acid. Cells were frozen in liquid nitrogen and disrupted with 0.08 g of glass beads by vortexing at 3,000 rpm for 10 min at 4°C. After dilution with 5% (w/v) metaphosphoric acid and centrifugation at 15,000 g for 10 min at 4°C, 50 μL of supernatants were mixed with 10 μL of 6 mM 2,6-dichlorophenolindophenol (DCPIP) and incubated for 20 min at room temperature. The sample was added with 25 μL of 2% (w/v) thiourea in 2% (w/v) metaphosphoric acid and with 12.5 μL of 2% (w/v) 2,4-dinitrophenylhydrazine in 25% (v/v) sulfuric acid. After incubation at 50°C for 1 h, the sample was added with 62.5 μL of 85% (v/v) sulfuric acid on an ice bath and measured at 520 nm.

### 2.4 Determination of the F_v_/F_m_ level

The F_v_/F_m_ level was measured using a MultispeQ v2.0 spectrophotometer (PhotosynQ) with a method described by Chiang et al. (2022) with minor modifications. In brief, cells were centrifuged at 1,000 × g for 6 min and resuspended in a fresh MA liquid medium with the Chl *a* concentration adjusted to 10 μg mL^−1^. The resuspended cells were incubated on a rotary shaker (125 rpm) in darkness at 40°C for 30 min to reach a dark-acclimated state. For each measurement, 1.5 mL of cells were placed in a 1 × 1-cm square cuvette clamped between the light guides. Fluorescence was recorded using an amber LED (30 ns × 400 μmol photons m^−2^ s^−1^) as measuring light source. A saturating light pulse (160 ms × 8000 μmol photons m^−2^ s^−1^, red LED) was used to probe the maximum fluorescence (F_m_) level. The F_v_/F_m_ level was calculated as (F_m_-F_o_)/F_m_, where F_o_ is the basal fluorescence level before the saturating light.

### 2.5 Determination of the activities of ascorbate-related enzymes

To extract enzymes, harvested cells were resuspended to the cell density equivalent to 4×10^9^ cells/mL in 50 μL of of extraction buffer containing 20% (v/v) glycerol, 5% (w/v) polyvinylpyrrolidone (PVP-40), 100 mM tricine-KOH (pH 7.8), 1 mM diethylenetriaminepentaacetic acid (DTPA) and 5 mM 2-mercaptoethanol (Smirnoff and Colombé, 1988). The cells were disrupted with 0.04 g of glass beads (0.5 mm in diameter) using a Bullet Blender Storm bead-mill homogenizer (Model BBY24M, Next Advance) at the speed set to 12 for two cycles of 5 min at 4°C. After centrifugation at 10,000 g for 1 min at 4°C, the supernatant was taken as the enzyme extract.

The SOD activity was determined using the superoxide dismutase activity assay kit (ab65354, Abcam) as per the manufacturer’s instruction. The APX and DHAR activities were measured based on the methods described by Smirnoff and Colombé (1988). The MDHAR and GR activities were assayed based on the methods described by Nakano and Asada (1981) and Mannervik (1999), respectively. To determine the APX activity, 10 μL of the enzyme extract was added with 200 μL of 50 mM potassium phosphate buffer (pH 7.0) mixed with 1 mM DTPA, 0.4 mM ascorbic acid, and 0.8 mM hydrogen peroxide. The APX activity was estimated based on the decay rate of ascorbic acid using the extinction coefficient of 6.15 mM^-1^ cm^-1^ at 285 nm. To determine the GR activity, 10 μL of the enzyme extract was added with 200 μL of reaction mixture (1 mM ethylenediaminetetraacetic acid (EDTA), 1 mM glutathione disulfide, 0.1 mM dihydronicotinamide-adenine dinucleotide phosphate (NADPH), 0.5 mM Tris-HCl in 100 mM potassium phosphate pH 7.0). To determine the MDHAR activity, 10 μL of the enzyme extract was added wtih 200 μL of reaction mixture (1 mM DTPA, 0.1 mM nicotinamide adenine dinucleotide (NADH), 2.5 mM ascorbic acid in 50 mM Tris-HCl pH 7.6), and ascorbate oxidase (1 unit/mL) (J65782, Alfa Aesar) was added just before absorbance measurement. Both GR and MDHAR activities were estimated from the oxidation rate of NAD(P)H using the extinction coefficient of 6.2 mM^-1^ cm^-1^ at 340 nm. To determine the DHAR activity, 10 μL of the enzyme extract was added with 200 μL of 50 mM potassium phosphate buffer (pH 7.0) mixed with 2.5 mM glutathione, 0.2 mM DHA, and 0.1 mM EDTA•2Na. The DHAR activity was estimated based on the production rate of ascorbic acid using the extinction coefficient of 14 mM^-1^ cm^-1^ at 265 nm.

### 2.6 Differential gene expression analysis

Light-dependent expression analysis of the genes encoding SOD, APX, MDHAR, GR, and L-galactono-1,4-lactone dehydrogenase (GLDH) was conducted using the existing RNA-seq data of *C. yangmingshanensis* grown in the dark, low light (20 μmol photons m^−2^ s^−1^) and high light (300 μmol photons m^−2^ s^−1^) for 6 d (SRA accessions: SRX6816975, SRX6816977, SRX6816979) (Liu et al., 2020). The RNA-seq reads were aligned to a combined genome sequence consisting of the nuclear, mitochondrial, and chloroplast genome sequences of *C. yangmingshanensis* (GenBank accessions: PRJNA564651, MN431656, and MN431657) using STAR (Dobin et al., 2013). Alignment-based transcript abundance was estimated using RSEM (Li and Dewey, 2011) and then normalized using the TMM (Trimmed Mean of M-values) method (Robinson and Oshlack, 2010). The resulting log_2_FC value of the gene of interest was used to calculate the difference between the log_2_FC values under the light (low light or high light) and dark conditions. The subcellular location of the gene was predicted based on the signal peptide identified using TargetP 2.0 (Almagro Armenteros et al., 2019), and the location was assigned as cytosol if no targeting peptide was identified.

## 3 Results

### 3.1 Changes in the cell density, the cellular contents of photosynthetic pigments, and the F_v_/F_m_ level under different light conditions

To characterize the photoacclimation response of *C. yangmingshanensis* under different light conditions, cells were transferred from the low light condition at 20 μmol photons m^–2^ s^–1^ into four light conditions: dark, low light (LL), medium light (ML), and high light (HL; equivalent to 0, 20, 200, and 1000 μmol photons m^–2^ s^–1^, respectively). The cell density, F_v_/F_m_, and cellular contents of Chl *a*, carotenoids, phycocyanin were measured.

**Figure 1:**
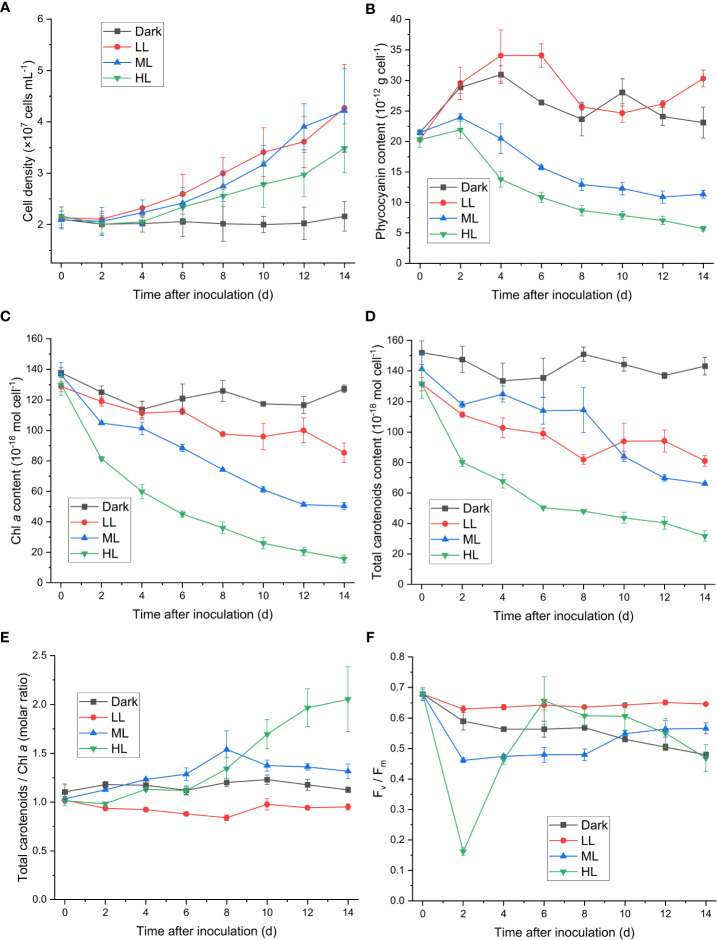
Effect of the light conditions on cell proliferation, changes in the photosynthetic pigments and in the F_v_/F_m_ value. Cells were inoculated to the cell density equivalent to 2×10^7^ cells mL^−1^ and grown at 40°C with shaking at 150 rpm in the dark, LL, ML, and HL conditions (equivalent to 0, 20, 200, and 1000 μmol photons m^−2^ s^−1^, respectively) for 14 d. Changes in the cell density **(A)**, the cellular content of phycocyanin **(B)**, the cellular content of Chl *a* **(C)**, the cellular content of total carotenoids **(D)**, the molar ratio of total carotenoids to Chl *a* **(E)**, and the F_v_/F_m_ value **(F)** were measured every 2 d. Data are expressed as average ± SD of three independent experiments.

Cell proliferations of *C. yangmingshanensis* under ML and LL conditions were indistinguishable and that under HL condition was relatively slow (**Figure 1A**). Nevertheless, cells still proliferated under HL condition for 14 d. The cellular contents of many photosynthetic pigments were significantly affected under ML and HL conditions compared with those under LL condition. The cellular content of phycocyanin, indicative of the amount of the light-harvesting phycobilisome, steadily decreased over time under ML and HL conditions, and the decrease in the phycocyanin level was stronger in HL than that in ML (**Figure 1B**). A similar trend of decrease was also observed in the cellular content of Chl *a* under ML and HL conditions (**Figure 1C**), indicating that the number of photosystems per cell decreased with increasing light intensities. Total carotenoids were decayed to various extents under different light conditions (**Figure 1D**). The decay of carotenoids was the strongest under HL. The cellular content of carotenoids under ML was higher than that under LL within 8 d and became lower than that under LL after 10 d. Despite variable extents of decrease in the number of carotenoids per cell, the relative number of carotenoids on Chl *a* basis displayed distinct trends under the three different light conditions. The ratio of carotenoids to Chl *a* increased under ML and HL conditions and slightly decreased under LL condition, and the ratio became prominently higher after 10 d under HL than that under ML and LL (**Figure 1E**).

Zeaxanthin, cryptoxanthin, and β-carotene are three carotenoid species identified in cyanidiophytes, with zeaxanthin and β-carotene present as the major forms. Both the zeaxanthin/Chl *a* ratio and cryptoxanthin/Chl *a* ratio similarly increased after cells transferred to HL condition, while the β-carotene/Chl *a* ratio began to increase after 6 d under HL (**Supplementary Figures 1A–C**). As a result, the zeaxanthin/β-carotene ratio under HL increased from 0 to 6 d and then decreased to a steady level after 10 d (**Supplementary Figure 1D**). Under ML condition, the zeaxanthin/Chl *a*, β-carotene/Chl *a*, and cryptoxanthin/Chl *a* ratios similarly increased from 0 to 8 d and then decreased after 8 d (**Supplementary Figures 1A–C**), and the zeaxanthin/β-carotene ratio slightly increased over time (**Supplementary Figure 1D**). The zeaxanthin/Chl *a*, β-carotene/Chl *a*, and cryptoxanthin/Chl *a* ratios were generally lower under LL than under dark condition (**Supplementary Figures 1A–C**). The zeaxanthin/β-carotene ratio under LL slightly increased up to 8 d and then decreased, and no apparent change in the zeaxanthin/β-carotene ratio was observed under dark condition (**Supplementary Figure 1D**).

The F_v_/F_m_ level reflects the maximum quantum efficiency of PSII photochemistry, and its decrease indicates photoinhibition the PSII activity. The F_v_/F_m_ level decreased within 2 d under ML and HL conditions (**Figure 1F**), and the rate and extent of decrease in F_v_/F_m_ were stronger under HL than that under ML (**Supplementary Figure 1**). The recovery of the PSII activity, as indicated by the increase of F_v_/F_m_, was observed from 2 to 6 d under ML and HL conditions, and the extent of the recovery was stronger under HL than that under ML (**Figure 1F**). This result suggested that photoacclimation response against the impairment of the PSII activity was slowly induced and became prominent after 2 d. The F_v_/F_m_ level further increased after 6 d under ML, while it steadily decreased after 6 d under HL. A steady decrease in F_v_/F_m_ was observed under dark condition, whereas change of F_v_/F_m_ was not observable under LL condition.

### 3.2 Accumulation of ascorbic acid under different light conditions

**Figure 2:**
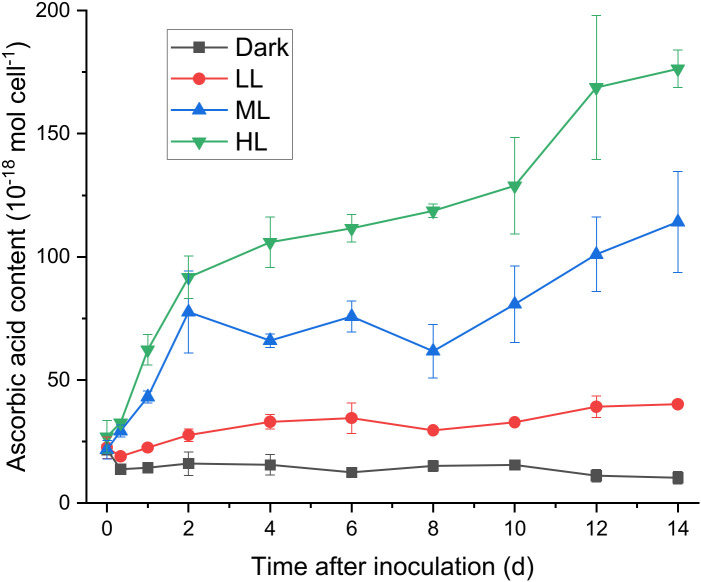
Effect of the light conditions on the cellular content of ascorbic acid. The growth condition was the same as described in **Figure 1**. Changes in the cellular content of ascorbic acid were measured at 0 h, 8 h, 1 d after inoculation and every 2 d after 2 d of photoacclimation. Data are expressed as average ± SD of at least three independent experiments.

To determine whether ascorbic acid accumulated in *C. yangmingshanensis* under illumination, the cellular content of ascorbic acid was measured. Ascorbic acid apparently accumulated under ML and HL conditions, and the overall accumulation of ascorbic acid was higher under HL than that under ML (**Figure 2**). By contrast, the cellular content of ascorbic acid increased only slightly under LL condition and did not increase under dark condition. Accumulation of ascorbic acid under HL and ML could be characterized by three phases based on the rate of change: a rapid phase within 2 d, a steady phase between 2-8 d, and a mild phase after 8 d. The cellular content of ascorbic acid increased under ML and HL during the rapid and mild phases and, during the steady phase, slightly increased under HL yet slightly decreased under ML. Interestingly, the period of 2 d in which a large amount of ascorbic acid was accumulated coincided with the period of 1-2 d after which the PSII activity was recovered (**Figure 1F** and **Supplementary Figure 2**), suggesting that the biosynthesis of ascorbic acid was enhanced to counteract light stress.

### 3.3 Activities of ascorbate-related ROS scavenging and regenerating enzymes under different light conditions

To investigate the involvement of the ascorbate-related enzymatic ROS scavenging process in photoacclimation, the activities of ascorbate-related ROS scavenging enzymes (SOD, APX), ascorbate regenerating enzymes (MDHAR, DHAR) and reduced glutathione regenerating enzyme (GR) were measured. The enzymatic activities were measured on the 2^nd^ and 6^th^ days during the light treatment, since the F_v_/F_m_ level strongly decreased within 2 d and then strongly increased from 2 to 6 d under HL (**Figure 1F**) while the cellular content of ascorbic acid drastically increased within 2 d yet became nearly steady between 2-6 d under the same condition (**Figure 2**).

**Figure 3:**
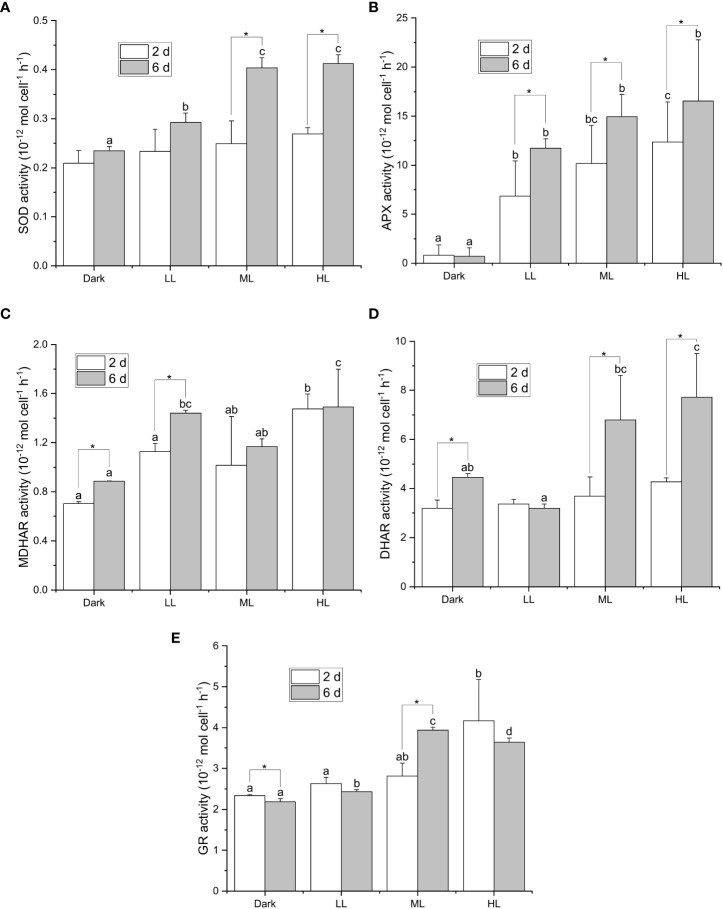
Effect of the light conditions on the activities of enzymes involved in the ascorbate-related enzymatic ROS scavenging system. Cells were inoculated to the cell density equivalent to 2×10^7^ cells mL^−1^ and grown at 40°C with shaking at 150 rpm in the dark, LL, ML, and HL conditions (equivalent to 0, 20, 200, and 1000 μmol photons m^−2^ s^−1^, respectively) for 6 d. Changes in the SOD activity **(A)**, APX activity **(B)**, MDHAR activity **(C)**, DHAR activity **(D)**, and GR activity **(E)**, were measured at 2 and 6 d after inoculation. Data are expressed as average ± SD of at least three independent experiments. Lowercase letters indicate significantly different groups of the same time as determined using one-way ANOVA followed by Tukey’s multiple comparison test, and the asterisks indicate a significance difference between 2 and 6 d under the same light condition using the two-sample *t*-test.

All the five enzymatic activities were enhanced in response to light, yet the onset and extent of enhancement differed under different light conditions (**Figure 3**). The light-dependent response of the SOD activity was observed only after 6 d under LL, ML, and HL conditions, and the SOD activity after 6 d was significantly higher under ML and HL than that under LL (**Figure 3A**). The APX activity was significantly enhanced by illumination after 2 and 6 d, and the extent of enhancement in the APX activity was dependent on the light intensity (**Figure 3B**). Furthermore, the APX activity significantly increased from 2 to 6 d under LL, ML, and HL conditions. The MDHAR activity was slightly induced by illumination, yet its dependency on the light intensity was less apparent (**Figure 3C**). The DHAR activity was strongly enhanced only after 6 d under ML and HL and was slightly enhanced under dark condition (**Figure 3D**). The GR activity was significantly enhanced after 6 d under ML and after 2 and 6 d under HL (**Figure 3E**). Neither DHAR activity nor GR activity enhanced remarkably under LL condition (**Figures 3D, E**). To sum up, the response of APX activity to the light intensity and the period of illumination turned out to be the most apparent among the five enzymatic activities.

### 3.4 Light-dependent expression of transcripts encoding ascorbate-related ROS scavenging and antioxidant regenerating enzymes

To evaluate whether the light-dependent activity of the ascorbate-related ROS scavenging or antioxidant regenerating enzyme could be reflected by the transcript level of the corresponding gene(s), differential expression analysis was conducted using the RNA-seq data of *C. yangmingshanensis* grown in the dark and illumination conditions. The light intensity of the high light condition for the RNA-seq data was different from those for the other experiments in this work (see Materials and Methods for details), yet differential expression analysis using these RNA-seq data still provided insight on the light-dependent transcriptional regulation. Furthermore, the organelle-specific activation of the ROS scavenging enzymes could be identified based on differentially expressed genes encoding the same enzyme yet targeted to different subcellular compartments. As shown in **Table 1**, three SOD and two APX genes targeted to different subcellular compartments were identified. Transcriptional expression of all the three SOD genes was apparently upregulated by illumination, and the chloroplast-targeted SOD gene was the highest upregulated gene among them. Transcriptional expression of the chloroplast-targeted APX gene was strongly upregulated by illumination, which was in sharp contrast to the unaltered expression of the cytosol-targeted counterpart. Light-induced expression of the chloroplast-targeted SOD and APX genes was associated with light-induced activities of the corresponding enzymes (**Figures 3A, B**), suggesting that the activities of the SOD and APX enzymes were dominantly enhanced in the chloroplast. The transcript encoding GR was also upregulated by illumination and associated with the light-induced GR activity (**Figure 3E**). Only one MDHAR gene targeted to the mitochondrion was identified, and its expression was not apparently induced by light. Furthermore, the transcript encoding GLDH, which catalyzed the terminal reaction of the *de novo* biosynthesis of ascorbic acid, was not apparently induced by light. No gene encoding DHAR was identified in the genome of *C. yangmingshanensis*.

**Table 1:** The log_2_ fold change (log_2_FC) value of transcripts encoding ascorbate-related ROS scavenging and regenerating enzymes.

UniProtKB Entry	Protein Name	Predicted subcellular location	log_2_FC		TMM-normalized expression value		
			Low light − Dark	High light − Dark	Dark	Low light	High light
A0A7J7IH89	Superoxide dismutase	Thylakoid lumen	5.61	5.31	8.74	473.96	385.36
A0A7J7IPC8	Superoxide dismutase	Mitochondrion	1.24	1.56	100.12	237.77	297.51
A0A7J7IID1	Superoxide dismutase	Cytosol	2.18	2.58	716.03	3253.09	4286.77
A0A7J7IJ03	Ascorbate peroxidase	Thylakoid lumen	4.57	5.27	219.01	5214.55	8510.23
A0A7J7IFJ8	Ascorbate peroxidase	Cytosol	-0.05	0.27	85.46	82.52	103.13
A0A7J7IS22	Monodehydroascorbate reductase	Mitochondrion	0.34	0.59	310.19	391.53	468.70
A0A7J7IGZ7	Glutathione reductase	Cytosol	3.05	2.86	40.03	338.20	296.75
A0A7J7IDS3	L-galactonolactone dehydrogenase	Cytosol	-0.41	0.03	97.20	73.02	99.50

The log_2_FC value was calculated as the difference between values under the illumination (low light or high light) and dark conditions; the subcellular location was assigned based on the prediction result using TargetP 2.0 (see Materials and Methods for details).

### 3.5 Effect of the APX inhibitors on photosynthesis under HL condition

**Figure 4:**
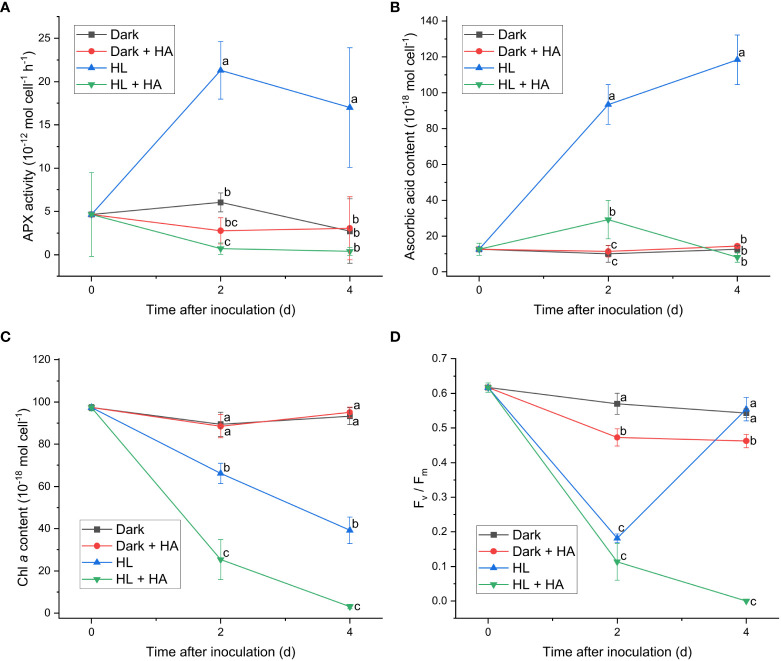
Effect of hydroxylamine on the APX activity **(A)**, the cellular content of ascorbic acid **(B)**, the cellular content of Chl *a* **(C)**, and the F_v_/F_m_ level **(D)**. Cells were inoculated to the cell density equivalent to 2×10^7^ cells mL^−1^ and grown at 40°C with shaking at 150 rpm in the dark and HL conditions (equivalent to 0 and 1000 μmol photons m^−2^ s^−1^, respectively) with and without addition of 10 mM of hydroxylamine (HA) for 4 d. Data are expressed as average ± SD of four independent experiments. Lowercase letters indicate significantly different groups of the same time as determined using one-way ANOVA followed by Tukey’s multiple comparison test.

**Figure 5:**
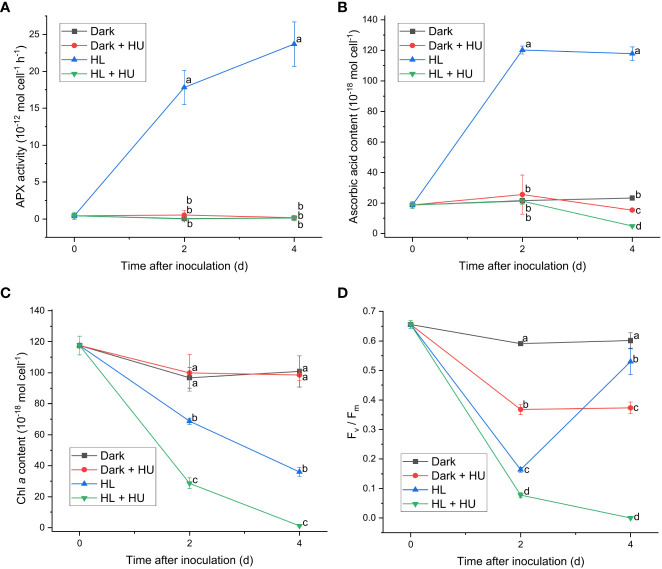
Effect of hydroxyurea on the APX activity **(A)**, the cellular content of ascorbic acid **(B)**, the cellular content of Chl *a* **(C)**, and the F_v_/F_m_ level **(D)**. Cells were inoculated to the cell density equivalent to 2×10^7^ cells mL^−1^ and grown at 40°C with shaking at 150 rpm in the dark and HL conditions (equivalent to 0 and 1000 μmol photons m^−2^ s^−1^, respectively) with and without addition of 25 mM of hydroxyurea (HU) for 4 d. Data are expressed as average ± SD of four independent experiments. Lowercase letters indicate significantly different groups of the same time as determined using one-way ANOVA followed by Tukey’s multiple comparison test.

Since APX exhibited the most distinct light-induced activity among the enzymatic activities measured in this work, two APX inhibitors, hydroxylamine (HA) and hydroxyurea (HU) (Chen and Asada, 1990), were utilized to assess the significance of the APX activity in photoacclimation. Photoacclimation for 4 d under HL was chosen for assessing the effect of HA and HU on photosynthesis, as HL led to the most drastic decrease and increase in the F_v_/F_m_ level within 4 d among the four light conditions (**Figure 1F**). The light-independent effect of HA and of HU on photosynthesis were assessed by measuring the APX activity, the F_v_/F_m_ level, and the Chl *a* content under dark condition with or without addition of HA or HU. The concentration of the APX inhibitors (10 mM for HA and 25 mM for HU) was chosen to maximize inhibition of the APX activity and to minimize undesired effects of the inhibitors. Both HA and HU significantly diminished the APX activity and unexpectedly diminished the cellular content of ascorbic acid at the same time (**Figures 4A, B**, **5A, B**). The decrease of the Chl *a* content under HL was accelerated by the two APX inhibitors, indicating that photoinhibition was stronger in the absence of APX activity and ascorbic acid than in their presence (**Figures 4C**, **5C**). Furthermore, the drastic recovery of the F_v_/F_m_ level after 4 d under HL was completely depleted by addition of HA or HU (**Figures 4D**, **5D**). Although these APX inhibitors mildly diminished the F_v_/F_m_ level in the dark condition possibly due to inhibition of oxygen evolution (see Discussion), no significant change in the Chl *a* content was observed in the dark condition (**Figures 4C, D**, **5C, D**). The overall effect of HA and HU on the F_v_/F_m_ level and the Chl *a* content under HL suggested that enhancement of the APX activity under illumination played a crucial role in photoacclimation.

## 4 Discussion

This work identified activation of the ascorbate-related enzymatic ROS scavenging system as an important photoacclimation response of *C. yangmingshanensis*. In this ROS scavenging system, the cellular content of ascorbic acid and the APX activity were apparently regulated by illumination. Light-dependent regulation of the APX activity was mediated by transcriptional regulation of the chloroplast-targeted APX gene. The inhibitory effect of the APX inhibitors on the PSII activity and the Chl *a* content under HL condition revealed that the APX activity played an important role in photoacclimation. In addition to the apparent increase in the ascorbic acid content and the APX activity, the cellular levels of the photosynthetic pigments steadily decreased under ML and HL conditions. Understanding the contribution of the ascorbate-related enzymatic ROS scavenging system to the protection of the photosynthetic apparatus allows us to probe further into how cyanidiophytes cope with photooxidative stress.

While accumulation of ascorbic acid was observed in *G. partita* as a response to high illumination under mixotrophic cultivation (Fu et al., 2020), it also served as a characteristic response to high illumination in *C. yangmingshanensis* under photoautotrophic cultivation. The maximum ascorbate amount per cell under HL (~1.75×10^-16^ mol cell^-1^) was equivalent to ~42 mM, assuming a sphere shape of the cell with a diameter of 2 μm based on the cell morphological features observed under microscope (Liu et al., 2020). This molar concentration was comparable to the ascorbate concentration (~40 mM) in the chloroplast of vascular plants under high illumination based on the immunocytochemical estimation (Zechmann et al., 2011). While both *C. yangmingshanensis* and *G. partita* displayed a similar extent of increase in the cellular content of ascorbic acid, the key regulation step for ascorbate biosynthesis at the transcriptional level appeared to be different. *C. yangmingshanensis* and *G. partita* possess GLDH and L-gulonolactone oxidase (GULO), respectively, as the terminal enzyme for ascorbate biosynthesis. Whereas the accumulation of ascorbic acid was associated with the upregulation of the transcript encoding GULO in *G. partita* (Fu et al., 2020), no apparent link between the cellular content of ascorbic acid and the number of transcript encoding GLDH was observed in *C. yangmingshanensis*. A similar phenomenon was observed in *Arabidopsis*, in which expression of the transcript encoding GLDH was not enhanced by illumination, while several transcripts encoding enzymes in the intermediate steps for ascorbate biosynthesis were upregulated (Yabuta et al., 2007). The utilization of a plant-like biosynthesis pathway of ascorbic acid in red algae was proposed (Wheeler et al., 2015), and it is possible that accumulation of ascorbic acid in *C. yangmingshanensis* is transcriptionally regulated at an intermediate step of ascorbate biosynthesis. However, the red algal genes encoding enzymes in the intermediate steps for ascorbate biosynthesis remain to be identified.

In addition to ascorbic acid, enzymes involved in ascorbate-related enzymatic ROS scavenging system also contribute to photoacclimation response in *C. yangmingshanensis*. The importance of ascorbic acid and APX in photoacclimation was revealed by the inhibitory effect on the PSII activity and the Chl *a* content in the presence of HA or HU. It was proposed that HA and HU radicals produced by APX inactivated the APX activity and could react with ascorbic acid (Chen and Asada, 1990). Depletion of ascorbic acid is thus likely attributed to that the oxidation rate of ascorbic acid reacting with HA or HU exceeds the recovery rate of ascorbic acid. An additional inhibitory effect of HA and HU on oxygen evolution in darkness and under illumination was reported (Cheniae and Martin, 1971; Kawamoto et al., 1994), providing a possible reason for the decreased F_v_/F_m_ level in the presence of HA or HU. It might be argued that inability to recover the PSII activity in the presence of HA or HU at 4 d under HL was attributed to accelerated destruction of the oxygen evolving complex under illumination, yet, on the contrary, oxygen evolution was reactivated by illumination in the presence of HA (Cheniae and Martin, 1972). Therefore, the light-dependent effect of HA and HU on the PSII activity at 4 d is most likely attributed to lack of the APX activity and the ascorbate content. Despite some undesired effects of HA and HU on targets other than APX, the importance of APX in photoacclimation can further be inferred from the APX activity drastically enhanced by illumination compared with that in darkness. Moreover, the APX activity significantly increased from 2 to 6 d of illumination under LL, ML, and HL conditions, while the cellular content of ascorbate acid did not apparently increase. A similar trend of increment in the SOD and DHAR activities from 2 to 6 d was also observed under ML and HL conditions. Notably, no gene encoding DHAR was identified in Cyanidiophyceae (Brawley et al., 2017 and the present work) while the DHAR activity was detected and regulated by light in *C. yangmingshanensis*. This suggests that the enzyme involved in reduction of DHA to regenerate ascorbic acid exists, and the gene responsible for such a reaction remains to be identified.

The involvement of APX in scavenging ROS produced in the chloroplast under illumination was supported by the result that only the APX gene targeted to the chloroplast was upregulated by light. The target site for the upregulated APX gene was initially predicted to be the thylakoid lumen using TargetP 2.0, yet proteins encoded by the homologous APX gene in *C. meraolae* were found in the stroma when this gene was expressed in *Arabidopsis* (Hirooka et al., 2009). The corresponding APX protein was therefore considered to be functional in the chloroplast instead of a more specific location of thylakoid lumen. The association of the light-dependent transcription of the APX gene targeted to the chloroplast with the light-dependent regulation of the APX activity suggested that the chloroplast APX activity was regulated at the transcriptional level. Inhibition of the chloroplast APX activity by H_2_O_2_ in the absence of ascorbic acid has been identified as another regulatory mechanism in plants, yet the algal APXs are generally tolerant to H_2_O_2_ (reveiwed in Caverzan et al., 2019). In *C. yangmingshanensis*, the APX activity was strongly enhanced with a relatively low ascorbic acid content under LL condition, supporting that the chloroplast APX activity was less likely to be regulated by H_2_O_2_.

Light-dependent accumulation of zeaxanthin in photosystem I was proposed as photoprotective mechanism for *C. merolae* based on the light-dependent increase in the zeaxanthin/Chl *a* ratio estimated in the cell and in the isolated photosystem I supercomplex (Haniewicz et al., 2018). Similarly, light-dependent increase in the zeaxanthin/Chl *a* ratio was observed under ML and HL conditions compared with the dark and LL conditions in *C. yangmingshanensis*. Although zeaxanthin might play a role in photoacclimation, the ascorbate-related enzymatic ROS scavenging system likely accounted for a major part of photoacclimation based on the time-course response of F_v_/F_m_, the ascorbic acid content, and the APX activity. Specifically, the recovery of the F_v_/F_m_ level began right after the drastic increase in the ascorbic acid content and the APX activity after 2 d of illumination under ML and HL. By contrast, the increase in the zeaxanthin/Chl *a* ratio was slow and continuous over 8 d and 14 d under ML an HL, respectively. Furthermore, the increment in the F_v_/F_m_ level from 8 to 14 d being stronger than that from 2 to 8 d under ML coincided with the corresponding increase in the ascorbic acid content from 8 to 14 d. By contrast, no apparent change in the zeaxanthin/Chl *a* ratio was observed from 8 to 14 d under the same light condition. The consistency between activation of the ascorbate-related enzymatic ROS scavenging system and the recovery of the PSII activity indicated the importance of this ROS scavenging system in photoacclimation.

Our data indicate that the APX activity and the transcript level of the chloroplast-targeted APX gene serve as markers of photoprotective processes against light stress in *C. yangmingshanensis*. Notably, some APX genes in *G. partita* were upregulated under illumination for 6 d with autotropic and heterotrophic cultivations, yet the extent of increase in these APX transcripts in *G. partita* was lesser than that in the APX transcript targeted to the chloroplast in *C. yangmingshanensis* (Fu et al., 2020). How the APX activity and transcript are regulated by light and associated with photoacclimation response in *G. partita* and other cyanidiophytes warrants further studies. Activation of the ascorbate-related ROS scavenging system, especially enhancement of the APX activity, provides a mechanistic explanation for the acclimation of *C. yangmingshanensis* to a wide range of light regimes in natural habitats. The importance of ascorbic acid and the APX activity in photoacclimation contributes to further optimization of growth conditions of the highly valued cyanidiophytes for biotechnological applications.

## Data availability statement

The datasets presented in this study can be found in online repositories. The names of the repository/repositories and accession number(s) can be found in the article/**Supplementary Material**.

## Author contributions

H-YF designed the experiments and drafted the manuscript. H-YF and M-WW performed the experiments, analyzed the data, and reviewed the manuscript. All authors contributed to the article and approved the submitted version.
