# Supplementary material for: Ascorbate peroxidase plays an important role in photoacclimation in the extremophilic red alga Cyanidiococcus yangmingshanensis
Source: Front Plant Sci. 2023 Jun 2;14:1176985. doi: 10.3389/fpls.2023.1176985 (PMC10272599; doi:10.3389/fpls.2023.1176985)
Supplement: Supplementary file 1 [file DataSheet_1.pdf]

Supplementary Material

Ascorbate peroxidase plays an important role in photoacclimation in the extremophilic red alga *Cyanidiococcus yangmingshanensis*

Han-Yi Fu*, Ming-Wei Wang

*** Correspondence:** Han-Yi Fu: hanyifu@mail.nsysu.edu.tw

1 Supplementary Figures

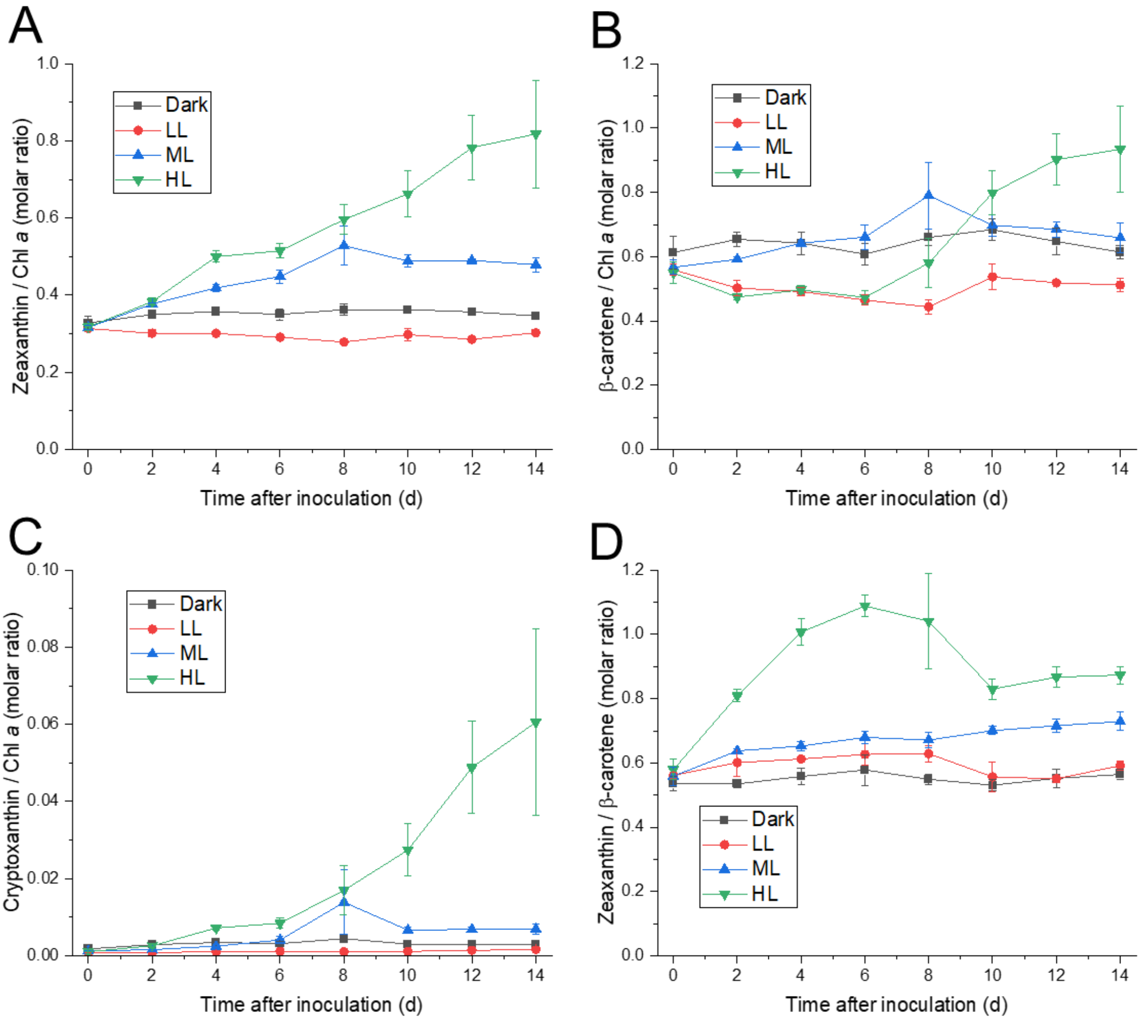

Supplementary Figure 1. Effect of the light conditions on the molar ratio of zeaxanthin to Chl *a* (A), β-carotene to Chl *a* (B), cryptoxanthin to Chl *a* (C), and zeaxanthin to β-carotene (D).

Cells were inoculated to the cell density equivalent to 2×10^{7} cells mL^{-1} and grown at 40°C with shaking at 150 rpm in the dark, LL, ML, and HL conditions (equivalent to 0, 20, 200, and 1000 µmol photons m^{-2} s^{-1}, respectively) for 14 d. Data are expressed as average ± SD of three independent experiments.

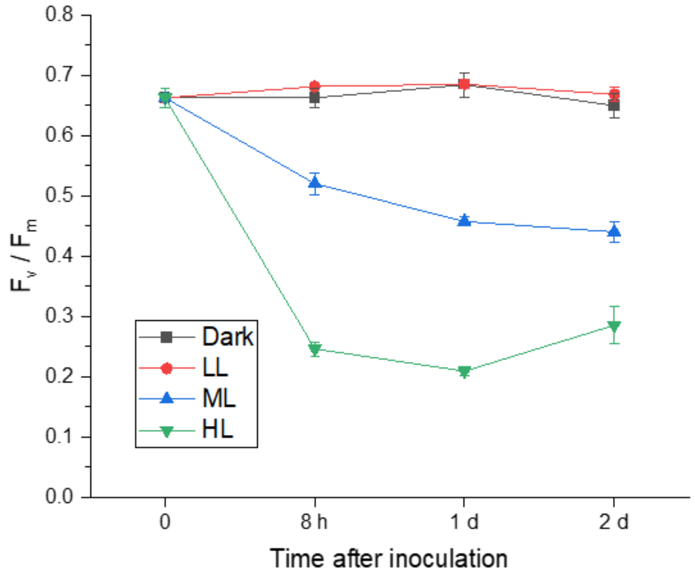

Supplementary Figure 2. Effect of the light conditions on decrease in the F_v/F_m value for 2 d.

Cells were inoculated to the cell density equivalent to 2×10^7 cells mL^{-1} and grown at 40°C with shaking at 150 rpm in the dark, LL, ML, and HL conditions (equivalent to 0, 20, 200, and 1000 μmol photons m^{-2} s^{-1}, respectively) for 2 d. Data are expressed as average ± SD of three independent experiments.
